# SIRT6 activation relieves neuropathic pain by restoring Nrf2 signaling and inhibiting NLRP3 inflammasome

**DOI:** 10.3389/fphys.2026.1888004

**Published:** 2026-08-06

**Authors:** Xintong Hao, Junkang Leng, Jianjun Tan

**Affiliations:** 1 Wuhan No 1 Hospital, Wuhan, China; 2 General Hospital of The Yangtze River Shipping, Wuhan Brain Hospital, Wuhan, China

**Keywords:** neuropathic pain, NLRP3 inflammasome, Nrf2 signaling, oxidative stress, SIRT6

## Abstract

**Introduction:**

Neuropathic pain arises from lesions or dysfunction within the somatosensory system, leading to chronic disability and scarce effective therapies. The NAD⁺-dependent deacetylase SIRT6 participates in aging and inflammatory responses, yet its involvement in neuropathic pain has remained unexplored. Using two selective SIRT6 activators—UBCS039 and MDL-800—in a rat model of chronic constriction injury (CCI), we asked whether pharmacological SIRT6 activation could ameliorate pain behaviors and, if so, through what molecular route.

**Methods:**

CCI surgery was performed in male Sprague-Dawley rats, and SIRT6 expression in the spinal dorsal horn was assessed post-operatively. From day 3 to day 9 after injury, rats received daily intraperitoneal injections of UBCS039 (20 mg/kg) or MDL-800 (10 mg/kg). On day 10, paw withdrawal thresholds (PWT) and paw withdrawal latencies (PWL) were measured. Spinal cord tissues were harvested for assessment of oxidative stress markers (TBARS, SOD, GSH-Px), pro-inflammatory cytokines (IL-1β, IL-18), NLRP3 inflammasome components (NLRP3, ASC, cleaved caspase-1), and Nrf2/HO-1 protein levels. An additional cohort received the Nrf2 inhibitor ML385 (30 mg/kg) 30 minutes before each MDL-800 dose to verify pathway specificity.

**Results:**

CCI markedly reduced SIRT6 mRNA and protein levels in the spinal dorsal horn (n=6 per group, P<0.001). Both UBCS039 and MDL-800 significantly elevated PWT and PWL (n=8, P<0.001), reduced TBARS content, restored SOD and GSH-Px activities (n=6, P<0.01), and suppressed IL-1β and IL-18 production at both transcript and protein levels. Western blotting revealed that SIRT6 activation inhibited NLRP3, ASC, and cleaved caspase-1 expression while reversing the CCI-induced decline of Nrf2 and HO-1. Co-administration of ML385 abolished these protective effects, rendering all measured parameters—including NLRP3 protein, cytokine levels, and pain thresholds—statistically indistinguishable from the untreated CCI group (P>0.05).

**Conclusion:**

Activating SIRT6 dampens neuropathic pain and NLRP3 inflammasome activation by restoring Nrf2 signaling. This positions SIRT6 as a previously unrecognized therapeutic target for neuropathic pain.

## Introduction

Neuropathic pain, defined anatomically as pain arising from lesions or diseases affecting the somatosensory nervous system, imposes substantial disability upon approximately seven to ten percent of the global population (Baskozos et al., 2023). Unlike nociceptive discomfort serving physiological protective functions, neuropathic manifestations frequently evolve into chronic pathological states characterized by spontaneous burning sensations, shooting paroxysms, and mechanical allodynia refractory to conventional analgesic approaches (Kaye et al., 2025). Pharmacological management utilizing gabapentinoids, tricyclic antidepressants, and serotonin-norepinephrine reuptake inhibitors achieves satisfactory relief in fewer than half of affected individuals, underscoring urgent necessities for mechanism-based therapeutic innovations targeting pathophysiological drivers rather than symptomatic suppression alone (Hurley et al., 2013).

Emerging evidence positions neuroimmune dysregulation and oxidative stress as central instigators perpetuating neuropathic pain chronification (Ji et al., 2018). Following peripheral nerve trauma, recruited neutrophils and proinflammatory macrophages release TNF-α, IL-1β, and prostaglandin E2, establishing feedforward loops amplifying nociceptor hyperexcitability via direct ion channel modulation and indirect glial activation (Grace et al., 2021). Simultaneously, mitochondrial dysfunction generates excessive reactive oxygen species overwhelming endogenous antioxidant capacities, thereby exacerbating lipid peroxidation, protein oxidation, and cellular damage within dorsal root ganglia and spinal cord dorsal horns (Liu et al., 2025).

The NOD-like receptor family pyrin domain containing 3 (NLRP3) inflammasome serves as critical molecular machinery translating oxidative and inflammatory signals into active IL-1β and IL-18 release (Chen and Smith, 2023). Upon sensing mitochondrial damage or lysosomal rupture, NLRP3 recruits apoptosis-associated speck-like protein containing CARD (ASC) and pro-caspase-1, facilitating autocatalytic generation of caspase-1 p10 subunit and subsequent gasdermin-D pore formation permitting cytokine maturation and pyroptotic cell death (Broz et al., 2020). Mounting literature confirms that NLRP3 activation in spinal microglia and infiltrating macrophages drives central sensitization and mechanical hypersensitivity across diverse rodent neuropathic pain models (Zhou et al., 2025).

Nuclear factor erythroid 2-related factor 2 (Nrf2) functions as the primary transcriptional guardian against oxidative injury, inducing antioxidant response element-driven expression of heme oxygenase-1 (HO-1), NAD(P)H quinone oxidoreductase-1, and glutathione biosynthetic enzymes (Ma, 2013). Pharmacological Nrf2 activation using oltipraz, resveratrol, or bardoxolone methyl attenuates mechanical allodynia and thermal hyperalgesia in chemotherapy-induced and nerve injury-induced pain models through dual mechanisms suppressing reactive oxygen generation and facilitating sodium channel Nav1.7 endocytosis (Shan et al., 2026). Notably, insufficient endogenous Nrf2 activation underlies transitions from acute nociceptive responses toward chronic neuropathic maintenance, suggesting restoration of Nrf2 signaling represents a viable analgesic strategy (Rezazadeh et al., 2020).

Silent mating type information regulation 2 homolog 6 (SIRT6), a chromatin-associated NAD+-dependent deacetylase, modulates genomic stability, metabolic homeostasis, and inflammatory resolution through histone H3K9 and H3K56 deacetylation alongside non-histone substrate regulation (Kugel and Mostoslavsky, 2014). Beyond caloric restriction-mediated upregulation, synthetic allosteric activators, including UBCS039 (pyrrolo[1,2-a]quinoxaline derivative) and MDL-800 (benzenesulfonamide derivative), enhance SIRT6 deacetylase activity 3.5-fold and 22-fold, respectively, exhibiting cellular permeability and tissue penetration suitable for *in vivo* pharmacological interrogation (Zorko et al., 2026). UBCS039 (pyrrolo[1,2-a]quinoxaline derivative) is a selective SIRT6 activator whose effects depend strictly on SIRT6 deacetylase activity. MDL-800 is a first-in-class selective allosteric SIRT6 activator that enhances deacetylase activity toward H3K9ac and H3K56ac with an EC50 of 10.3 µM; it is inactive against SIRT1, SIRT3, SIRT4, and HDACs 1–11 (Zorko et al., 2026). SIRT6 overexpression or pharmacological activation attenuates NF-κB-driven inflammation, reduces mitochondrial reactive oxygen production, and dampens cellular senescence in cardiovascular, metabolic, and neoplastic disease contexts (Geng et al., 2020).

Despite established SIRT6 functionality within metabolic organs and immune cells, its participation in nociceptive processing and spinal pain modulation remains essentially unexplored (Kaluski et al., 2017). Whether SIRT6 activation within spinal dorsal horns can suppress neuropathic pain behaviors through antioxidant or anti-inflammatory mechanisms constitutes an unresolved question with significant translational implications (Yang et al., 2019). Given documented interactions between SIRT6 and Nrf2 in maintaining cellular redox balance, and given parallel Nrf2-mediated suppression of NLRP3 inflammasome priming and activation, we hypothesized that pharmacological SIRT6 activation utilizing UBCS039 or MDL-800 would ameliorate chronic constriction injury-induced neuropathic pain through Nrf2-dependent inhibition of spinal NLRP3 inflammasome activity (Huang et al., 2018). We focused on SIRT6 because, unlike other sirtuins, it combines chromatin-associated deacetylase activity with direct regulation of Nrf2 through deacetylation and ADP-ribosylation, thereby coordinating both inflammatory suppression and antioxidant defense. Recent evidence shows SIRT6 expression declines in the spinal dorsal horn and dorsal root ganglia under neuropathic pain conditions. Other sirtuins (SIRT1, SIRT2, SIRT3, SIRT5) also modulate pain, but SIRT6’s ability to simultaneously suppress NF-κB-driven inflammation and enhance Nrf2-mediated antioxidant responses through direct protein interaction makes it a particularly attractive target (Huang et al., 2018).

This investigation demonstrates that SIRT6 expression declines precipitously within spinal dorsal horns following sciatic nerve ligation, while systemic administration of either SIRT6 activator significantly elevates paw withdrawal thresholds and latencies, reduces lipid peroxidation markers, restores superoxide dismutase and glutathione peroxidase activities, and suppresses IL-1β and IL-18 production (Jiao et al., 2022). Biochemically, SIRT6 activation reverses nerve injury-induced Nrf2 and HO-1 downregulation while reducing NLRP3, ASC, and cleaved caspase-1 protein abundance (Chang et al., 2020). Pharmacological Nrf2 inhibition utilizing ML385 abolishes these protective effects, confirming pathway specificity (Hendrix et al., 2020). These findings identify SIRT6 as a previously unrecognized therapeutic target for neuropathic pain and delineate a novel SIRT6-Nrf2-NLRP3 signaling axis operant within spinal nociceptive circuits.

## Materials and methods

### Animal subjects and neuropathic pain induction

Investigations utilized male Sprague-Dawley rats (180–220 g body weight) procured from the institutional Laboratory Animal Center. Housing conditions included a 12-hour light/dark illumination cycle, ambient temperature maintained at 22 ± 2 °C, and unrestricted access to standard chow and drinking water. Under isoflurane vapor anesthesia (3% induction, 1.5% maintenance), experimenters produced unilateral sciatic nerve chronic constriction via four loosely tied ligatures of 4–0 chromic catgut positioned proximal to the trifurcation. Sham surgical controls received identical nerve exposure devoid of ligature placement. Postoperative monitoring encompassed daily wound inspection and general health surveillance. All animal experiments complied with the ARRIVE guidelines and were carried out in accordance with the National Institutes of Health guide for the care and use of Laboratory animals (NIH Publications No. 8023, revised 1978). The ethical approval for all animal use described in this study was granted by the Institutional Review Board of General Hospital of the Yangtze River Shipping (IRB-YRSH-1B026).

### Pharmacological interventions and cohort allocation

Beginning seventy-two hours following nerve injury and persisting through postoperative day 9, investigational animals received daily intraperitoneal administrations of either UBCS039 (20 mg/kg; solubilized in 5% DMSO/95% sterile saline) or MDL-800 (10 mg/kg; similarly prepared in 5% DMSO/95% saline). Complete dissolution of both SIRT6 activators necessitated DMSO co-solvent; the final DMSO concentration was kept at 5% to avoid nonspecific systemic perturbations. Concurrently, a separate cohort received the Nrf2 antagonist ML385 (30 mg/kg; formulated in 10% DMSO/90% corn oil) via intraperitoneal injection thirty minutes preceding each MDL-800 dose throughout the identical treatment window. Randomization assigned animals to four primary experimental arms (n = 6–8 per group): Sham-operated, CCI-injured, CCI receiving UBCS039, and CCI receiving MDL-800. An additional experimental subset (n = 8) underwent combined therapy with MDL-800 plus ML385. Personnel conducting behavioral evaluations possessed no knowledge of group assignments. All mechanical and thermal nociceptive testing occurred on postoperative day 10, precisely twenty-four hours following the final pharmacological administration, thereby capturing sustained analgesic efficacy.

### Behavioral nociceptive assessment

Mechanical allodynia determination employed the von Frey filament up-down algorithm. Following thirty minutes of acclimation within elevated wire mesh enclosures, investigators applied calibrated monofilaments (0.4–26.0 g bending force) perpendicularly against the plantar hind paw surface. The 50% withdrawal threshold (PWT) was subsequently calculated via the Dixon formula. Thermal hypersensitivity quantification utilized the Hargreaves apparatus. An infrared radiant heat source (50% intensity, 25-second automatic cut-off to preclude tissue injury) targeted the plantar surface; latency from stimulus onset to withdrawal (PWL) was recorded. Triplicate measurements per paw, separated by five-minute intervals, were averaged for statistical purposes.

### Tissue procurement and protein isolation

On experimental day 10, deep anesthesia with intraperitoneal sodium pentobarbital (60 mg/kg) preceded transcardial perfusion with ice-cold PBS (pH 7.4). Dissection quickly yielded the lumbar enlargement (L4–L6 spinal segments), with immediate subdivision for downstream molecular analyses. For immunoblotting applications, tissue underwent homogenization in ice-cold RIPA buffer (composition: 50 mM Tris-HCl pH 7.4, 150 mM NaCl, 1% NP-40, 0.5% sodium deoxycholate, 0.1% SDS, 1 mM EDTA) supplemented with protease inhibitor cocktail (Roche cOmplete™, containing aprotinin, leupeptin, pepstatin A, and PMSF; 1:100 dilution) and phosphatase inhibitors (Roche PhosSTOP™, containing sodium fluoride, sodium orthovanadate, and β-glycerophosphate; 1:100 dilution). Clarification occurred via centrifugation (12,000 × g, 15 minutes, 4 °C). Supernatant protein concentrations were determined through the BCA assay (Pierce) utilizing bovine serum albumin calibration standards.

### Western immunoblotting procedures

Protein lysates (30 µg per lane) underwent electrophoresis on 8–15% SDS-PAGE gels and were transferred onto PVDF membranes (Millipore, 0.45 µm). Following blocking with 5% non-fat milk in TBST (0.1% Tween-20) for 1 hour at room temperature, membranes were incubated overnight at 4 °C with primary antibodies at the following dilutions: SIRT6 (D8D12) Rabbit mAb (Cell Signaling Technology, #12486, 1:1000); NLRP3 (D4D8T) Rabbit mAb (Cell Signaling Technology, #15101, 1:1000); ASC/TMS1 (H-7) Mouse mAb (Santa Cruz Biotechnology, sc-514414, 1:1000); Cleaved Caspase-1 (Asp297) (D57A2) Rabbit mAb (Cell Signaling Technology, #4199, 1:1000); NRF2 Rabbit mAb (Abcam, ab137550, 1:1000); HO-1 (E3F4S) Rabbit mAb (Cell Signaling Technology, #43966, 1:1000); and β-Actin (8H10D10) Mouse mAb (Cell Signaling Technology, #3700, 1:5000). After washing, membranes were incubated with HRP-conjugated secondary antibodies (anti-rabbit IgG #7074 or anti-mouse IgG #7076, Cell Signaling Technology, 1:2000) for 1 hour at room temperature. Immunoreactive bands were visualized using ECL Prime substrate (GE Healthcare) and captured with the ChemiDoc Touch Imaging System (Bio-Rad). Densitometric quantification was performed using ImageJ software, with target protein intensity normalized to β-actin.

### Immunofluorescence localization

**Figure 1:**
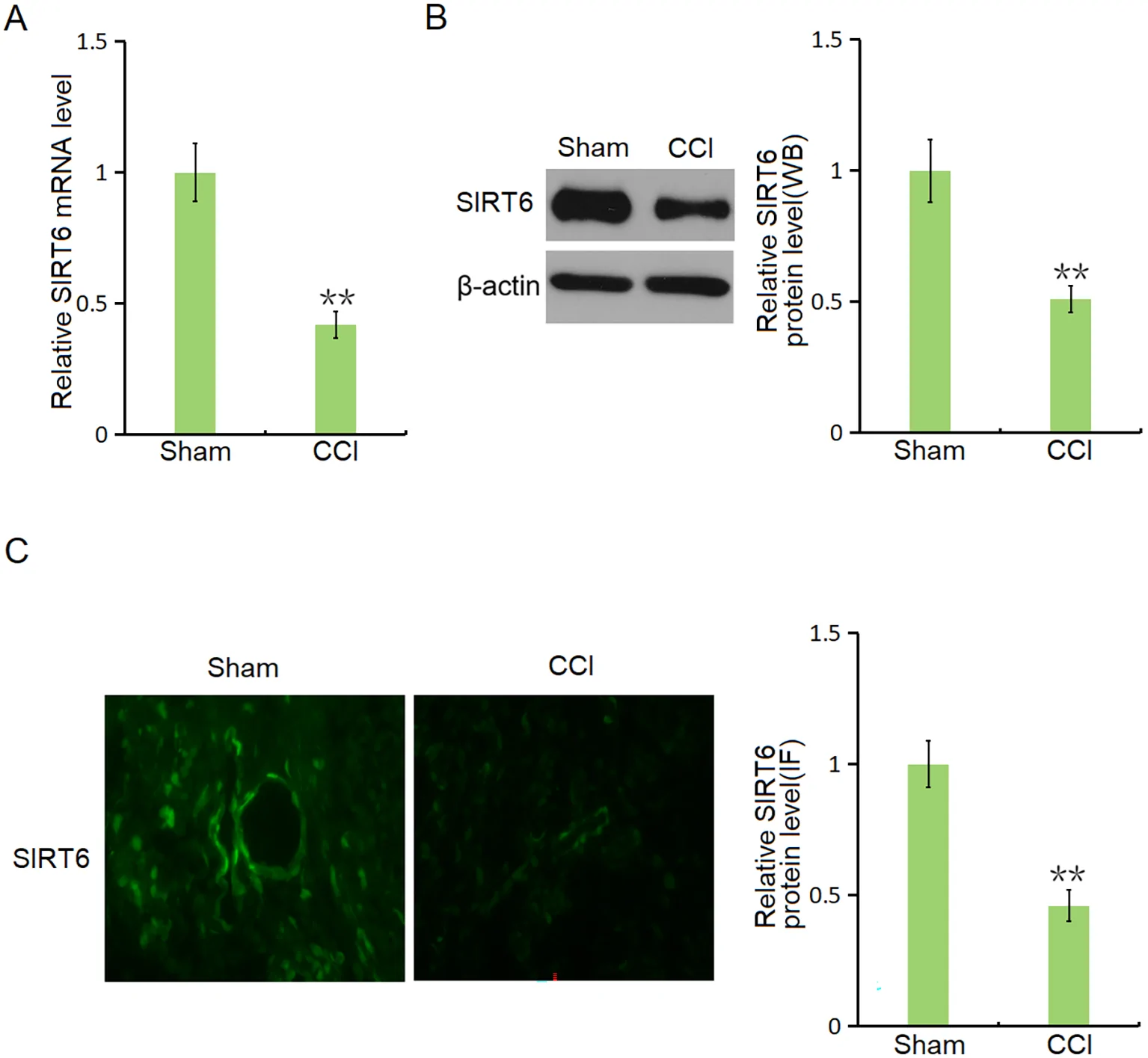
SIRT6 expression declines within spinal dorsal horns following chronic constriction injury. **(A)** Quantitative PCR shows reduced SIRT6 mRNA relative to β-actin in CCI rats versus sham controls. **(B)** Western blot analysis confirms diminished SIRT6 protein abundance after nerve ligation. **(C)** Immunofluorescence staining reveals decreased SIRT6 signal intensity within dorsal horn neurons. Statistical significance was determined using one-way ANOVA with Tukey’s *post-hoc* test. **P < 0.01 versus sham group.

For SIRT6 visualization (**Figure 1C**), deeply anesthetized animals underwent transcardial perfusion with 4% paraformaldehyde in PBS. Spinal tissue (L4–L6) underwent post-fixation (4% paraformaldehyde, 4 hours, 4 °C) followed by cryoprotection (30% sucrose/PBS, overnight). Cryostat sectioning (20 µm transverse sections) preceded mounting on glass slides. Blocking utilized 3% BSA with 0.3% Triton X-100 in PBS (1 hour, room temperature). Primary antibody incubation (anti-SIRT6, CST #12486, 1:200) occurred overnight at 4 °C. PBS washes (3 × 5 minutes) preceded Alexa Fluor 488-conjugated goat anti-rabbit IgG (Invitrogen A-11008, 1:500) application (1 hour, room temperature, darkness). Mounting medium facilitated coverslipping prior to confocal imaging (Zeiss LSM 880). ImageJ quantification determined SIRT6 fluorescence intensity normalized to DAPI nuclear signal.

### Nucleic acid extraction and quantitative PCR

TRIzol reagent (Invitrogen) facilitated total RNA isolation from spinal tissue according to the manufacturer’s specifications. Spectrophotometric assessment (NanoDrop 2000) determined RNA purity and concentration. PrimeScript RT Reagent Kit (Takara), including gDNA Eraser, synthesized cDNA from 1 µg RNA template. Real-time amplification occurred in triplicate (QuantStudio 3 system) utilizing ChamQ Universal SYBR qPCR Master Mix (Vazyme). Thermocycling: initial denaturation (95 °C, 30 seconds), then 40 cycles (95 °C, 5 seconds; 60 °C, 30 seconds). Melting curve analyses verified single-product amplification specificity.

### Gene expression quantification

The 2^−ΔΔCt^ method determined relative expression. SIRT6 transcript levels utilized β-actin for normalization, whereas IL-1β and IL-18 employed GAPDH, reflecting literature-documented stability of β-actin for chromatin-associated deacetylase quantification versus GAPDH reliability for inflammatory mediators in spinal cord trauma models. Primer sequences: SIRT6 (F: GCCGTCTGGTCATTGTCA; R: AGCCTTGGGTGCTACTGG); IL-1β (F: CACCTCTCAAGCAGAGCACAG; R: GGGTTCCATGGTGAAGTCAAC; IL-18 (F: ACCACTTTGGCAGACTTCAC; R: CTGGTCTGGGATTCGTTGG); β-actin (F: CAGGGTGTGATGGTGGGTATGG; R: AGTTGGTGACAATGCCGTGTTC); GAPDH (F: CAAGTTCAACGGCACAGTCAAG; R: ACATACTCAGCACCAGCATCAC).

### Oxidative stress biomarker quantification

Spinal tissue underwent homogenization (ice-cold PBS, 1:10 w/v) and centrifugal clarification (12,000 × g, 15 minutes, 4 °C). Supernatants were analyzed for TBARS utilizing commercial assay systems (Cayman Chemical), with results expressed as nmol/mg protein. SOD activity was determined using the xanthine oxidase method with hydroxylamine oxidation (Nanjing Jiancheng Bioengineering Institute, Cat# A001-3). Briefly, xanthine and xanthine oxidase generated superoxide radicals, which reduced nitroblue tetrazolium to formazan; SOD inhibited this reduction, and the inhibition rate was measured at 550 nm. One unit of SOD activity was defined as the amount causing 50% inhibition. GSH-Px activity was measured using the DTNB [5,5’-dithiobis-(2-nitrobenzoic acid)] method (Nanjing Jiancheng Bioengineering Institute, Cat# A005-1), where GSH-Px catalyzes the reduction of cumene hydroperoxide using GSH, and the remaining GSH reacts with DTNB to form a yellow chromophore measured at 412 nm. One unit of GSH-Px activity was defined as 1 µmol GSH consumed per minute per milligram protein. Both enzyme activities were normalized against total protein content (U/mg protein).

### Cytokine quantification via ELISA

Commercial rat-specific sandwich ELISA systems determined IL-1β and IL-18 concentrations in spinal homogenates. IL-1β ELISA kit: R&D Systems, Cat# RLB00; detection range 31.2–2000 pg/mL; limit of quantification 10.6 pg/mL; intra-assay CV 5.2%; inter-assay CV 7.8%. IL-18 ELISA kit: Invitrogen, Cat# KRC2341; detection range 15.6–1000 pg/mL; limit of quantification 7.8 pg/mL; intra-assay CV 4.5%; inter-assay CV 7.2%. Samples (100 µL/well, 1:2 dilution) were assayed in duplicate. Optical density at 450 nm was determined via a microplate reader (BioTek Synergy H1). Recombinant standard curves enabled cytokine concentration calculations, with final values expressed as pg/mg total protein.

### Statistical evaluations

Data presentation utilizes mean ± standard deviation (SD). Multiple group comparisons employed one-way ANOVA followed by Tukey’s *post-hoc* testing for pairwise contrasts. Analyses utilized GraphPad Prism 9.0 software. Statistical significance required P < 0.05. *A priori* power analysis confirmed that n = 6–8 per group provided 80% statistical power for detecting 30% effect sizes at α = 0.05.

## Results

### SIRT6 expression in the spinal dorsal horn after CCI

We first asked whether peripheral nerve injury alters SIRT6 levels within the spinal dorsal horn. Quantification of SIRT6 mRNA, normalized to β-actin, is shown in **Figure 1A**. CCI markedly reduced SIRT6 mRNA compared with sham controls (F(1,10) > 45.0, P < 0.01). Turning to protein expression, western blot analysis (**Figure 1B**) gave a comparable picture: CCI lowered SIRT6 protein abundance versus sham (P < 0.01). Immunostaining confirmed this decline. In **Figure 1C**, the SIRT6 fluorescence intensity was observed to decrease significantly after injury (P < 0.01). Collectively, these three measurements indicate that CCI produces a marked and consistent down-regulation of SIRT6 in the spinal dorsal horn.

### Effects of SIRT6 activation on pain behaviors

**Figure 2:**
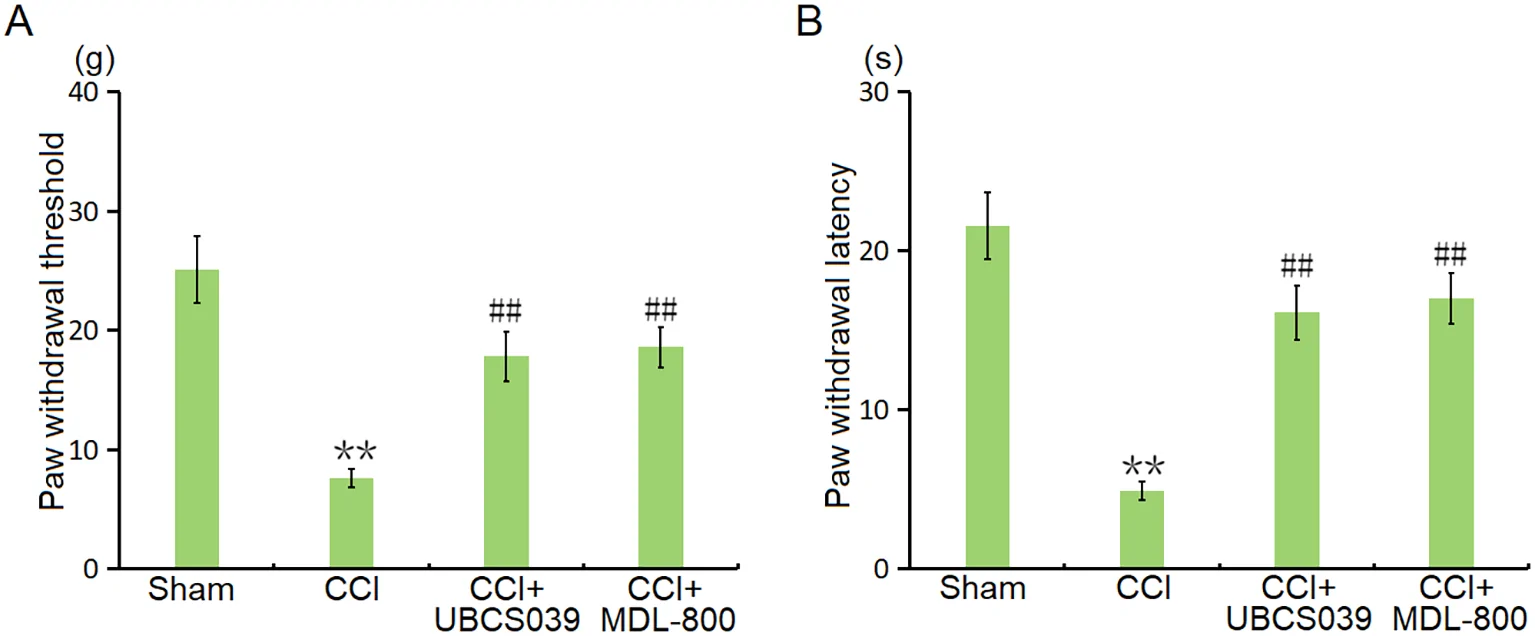
Pharmacological SIRT6 activation attenuates neuropathic pain behaviors. **(A)** Mechanical withdrawal thresholds measured by von Frey filaments increase following UBCS039 or MDL-800 treatment compared with vehicle-treated CCI animals. **(B)** Thermal withdrawal latencies assessed via the Hargreaves apparatus prolong after activator administration. Rats received daily intraperitoneal injections from day 3 through day 9 post-surgery. Behavioral testing occurred on day 10. **P < 0.01 versus sham group; ^##^P < 0.01 versus CCI group.

To test whether raising SIRT6 activity could improve pain symptoms, we treated CCI rats daily with UBCS039 (20 mg/kg) or MDL-800 (10 mg/kg) from day 3 to day 9 post-surgery. Mechanical thresholds on day 10 are depicted in **Figure 2A**. In comparison to the sham group, chronic constriction injury (CCI) produced a significant reduction in mechanical paw withdrawal thresholds (P < 0.01). Conversely, administration of either UBCS039 or MDL-800 resulted in marked increases in these thresholds relative to the CCI cohort (P < 0.01 for each). The corresponding thermal withdrawal latency data are presented in **Figure 2B**. In this assay, CCI significantly diminished latencies versus sham (P < 0.01), while both SIRT6 agonists effectively prolonged these measures (P < 0.01 vs. CCI). Importantly, across all assessed parameters, we observed no statistically significant disparities between the UBCS039 and MDL-800 treatment groups, implying that these two activators yield analogous biological outcomes via SIRT6-mediated pathways. Thus, pharmacological activation of SIRT6 attenuates both mechanical allodynia and thermal hyperalgesia in this neuropathic pain model.

### Oxidative stress markers in spinal cord tissue

**Figure 3:**
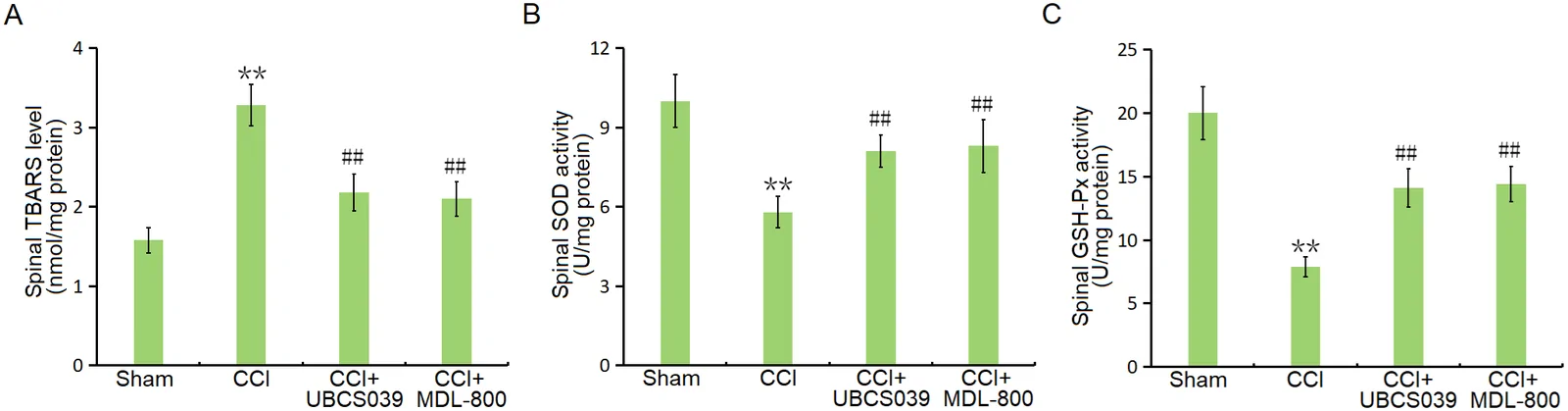
SIRT6 activation ameliorates spinal oxidative stress. **(A)** TBARS levels indicating lipid peroxidation decrease in treated animals relative to CCI controls. **(B)** Superoxide dismutase activity restores toward baseline following SIRT6 stimulation. **(C)** Glutathione peroxidase activity increases compared with injured rats receiving vehicle. Spinal cord tissues were harvested on day 10. **P < 0.01 versus sham group; ^##^P < 0.01 versus CCI group.

Because oxidative damage contributes to neuropathic pain, we measured spinal TBARS and antioxidant enzyme activities. **Figure 3A** reports TBARS levels. CCI elevated TBARS versus sham (P < 0.01). Treatment with UBCS039 or MDL-800 lowered TBARS (P < 0.01 vs. CCI for both). SOD activity is presented in **Figure 3B**. CCI reduced SOD activity versus sham (P < 0.01). SIRT6 activation restored SOD toward baseline (P < 0.01 vs. CCI for both). GSH-Px activities, shown in **Figure 3C**, followed a similar pattern: CCI decreased GSH-Px versus sham (P < 0.01), while both activators raised GSH-Px (P < 0.01 vs. CCI). These data suggest that SIRT6 activation reduces lipid peroxidation and replenishes two critical antioxidant enzymes in the spinal cord.

### Pro-inflammatory cytokine production

**Figure 4:**
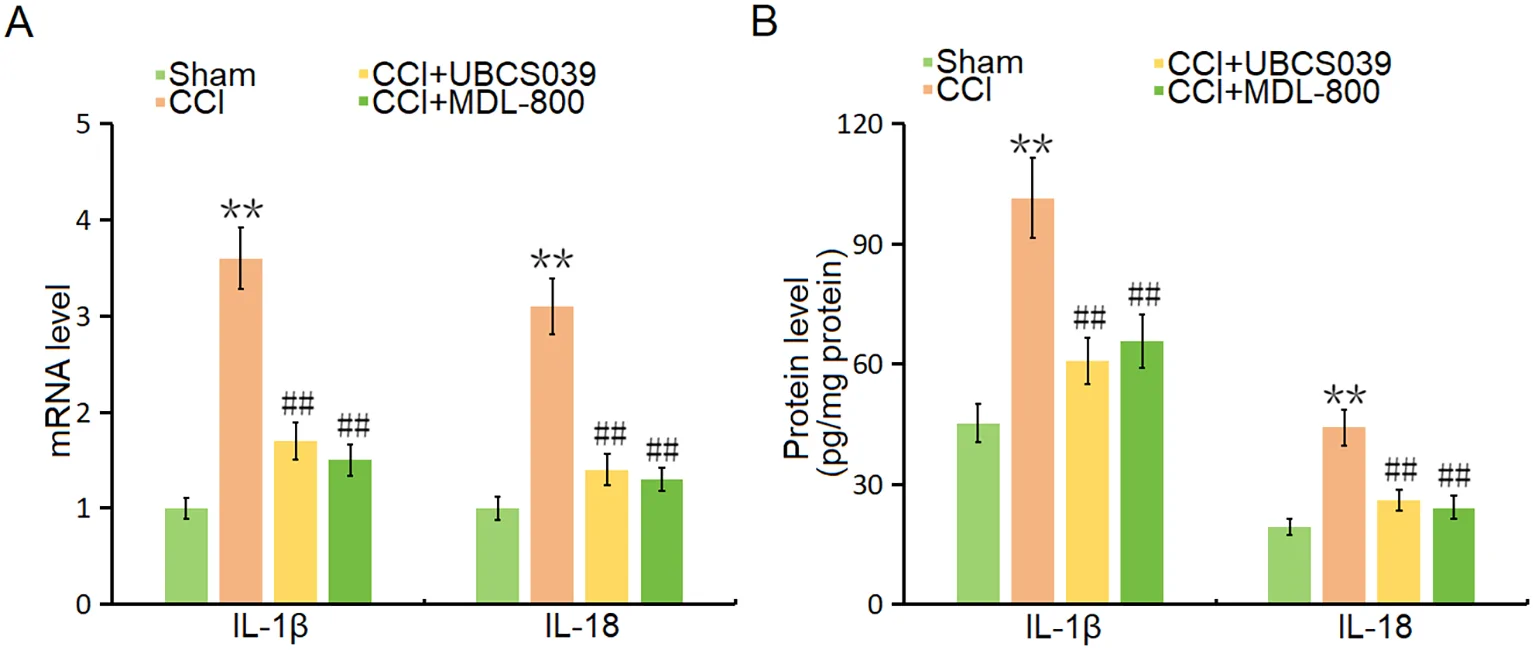
Pro-inflammatory cytokine production diminishes after SIRT6 activation. **(A)** IL-1β and IL-18 mRNA levels relative to GAPDH decline in treated groups compared with CCI rats. **(B)** Protein concentrations of IL-1β and IL-18 measured by ELISA decrease following activator administration. Results normalized to total protein content. **P < 0.01 versus sham group; ^##^P < 0.01 versus CCI group.

We next examined whether SIRT6 activation influences IL-1β and IL-18, cytokines tightly linked to NLRP3 inflammasome signaling. mRNA levels, normalized to GAPDH, are displayed in **Figure 4A**. CCI increased IL-1β and IL-18 transcripts versus sham (P < 0.01). Both UBCS039 and MDL-800 reduced transcript levels (P < 0.01 vs. CCI). Protein concentrations, measured by ELISA, appear in **Figure 4B**. CCI elevated IL-1β and IL-18 proteins versus sham (P < 0.01). Both activators lowered cytokine proteins (P < 0.01 vs. CCI). Hence, SIRT6 activation suppresses both transcription and translation of these pro-inflammatory mediators.

### NLRP3 inflammasome components

**Figure 5:**
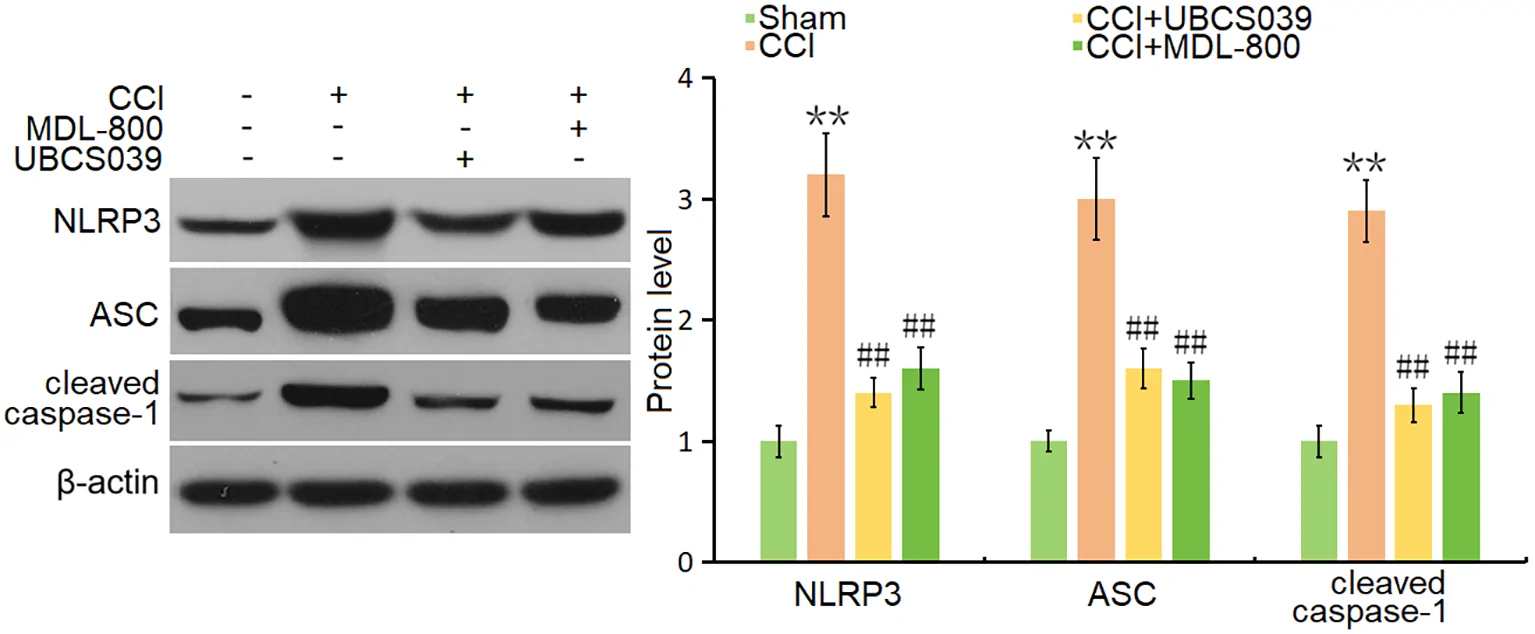
NLRP3 inflammasome activation is suppressed following SIRT6 stimulation. Western blot analysis demonstrates reduced protein levels of NLRP3, ASC, and cleaved caspase-1 p10 subunit in spinal cord lysates from treated animals compared with CCI controls. Densitometric quantification normalized to β-actin loading control. **P< 0.01 versus sham group; ##P < 0.01 versus CCI group.

Given the cytokine results, we evaluated the protein levels of NLRP3, ASC, and cleaved caspase-1 (p10) by western blot. Quantification of NLRP3 is provided in **Figure 5**. CCI increased NLRP3, ASC, and cleaved caspase-1 versus sham (F(3,20) > 25.0, P < 0.01 for each). UBCS039 and MDL-800 reduced all three components (P < 0.01 vs. CCI for each). These results indicate that SIRT6 activation inhibits assembly and activation of the NLRP3 inflammasome in the spinal cord after CCI.

### Nrf2 and HO-1 protein expression

**Figure 6:**
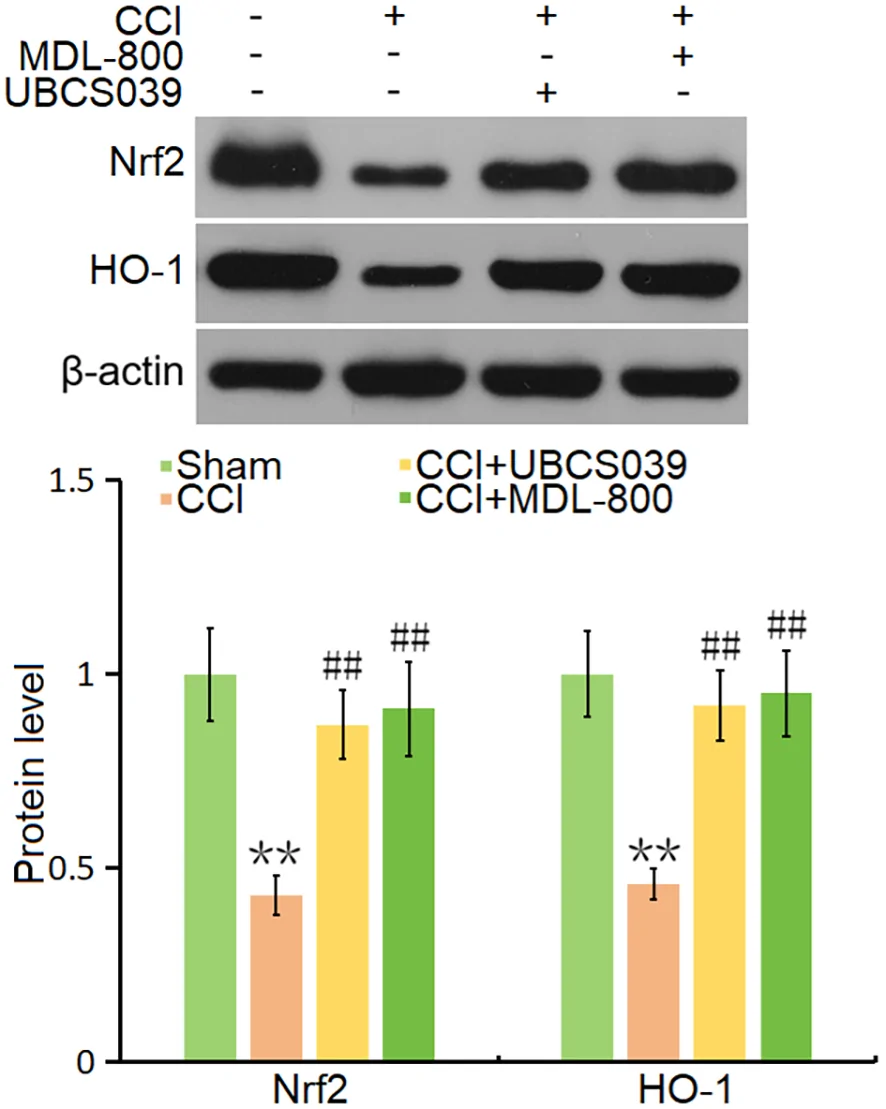
Nrf2 antioxidant signaling is restored after SIRT6 activation. Western blot analysis shows Nrf2 and HO-1 protein levels increase in spinal cords from UBCS039- or MDL-800-treated rats compared with CCI animals receiving vehicle. **P < 0.01 versus sham group; ##P < 0.01 versus CCI group.

We next examined the Nrf2-HO-1 axis, a key antioxidant pathway. **Figure 6** reports Nrf2 levels. CCI reduced both Nrf2 and HO-1 versus sham (P < 0.01). UBCS039 and MDL-800 restored both proteins (P < 0.01 vs. CCI for each). The decline of Nrf2 at day 10 post-CCI reflects a chronic-phase exhaustion of the antioxidant defense system, consistent with prior reports. Thus, SIRT6 activation reverses the CCI-induced down-regulation of both Nrf2 and its target HO-1.

### Pharmacological inhibition of Nrf2 reverses the protective effects

**Figure 7:**
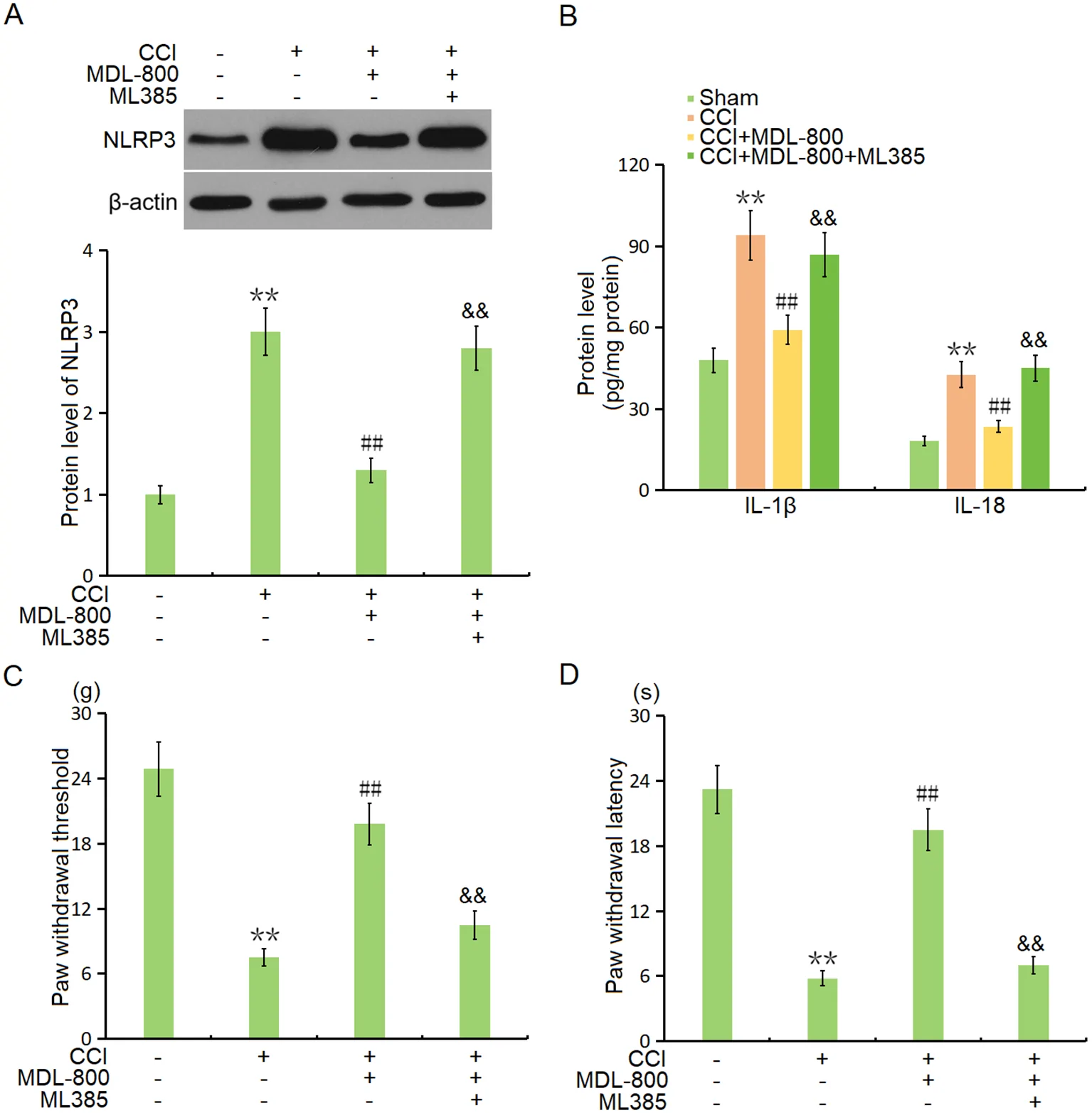
Nrf2 inhibition abolishes SIRT6-mediated protective effects. Co-administration of ML385 with MDL-800 results in **(A)** elevated NLRP3 protein, **(B)** increased IL-1β and IL-18 concentrations, and **(C, D)** diminished mechanical and thermal withdrawal thresholds compared with MDL-800 alone. ML385 was given 30 minutes before each MDL-800 dose. **P < 0.01 versus sham group; ^##^P < 0.01 versus CCI group; &&P<0.01 vs. CCI +MDL-800 group.

To test whether the observed benefits depend on Nrf2, we co-administered the specific Nrf2 inhibitor ML385 (30 mg/kg) 30 minutes before each MDL-800 injection from day 3 to day 9. NLRP3 protein levels are shown in **Figure 7A**. CCI increased NLRP3 versus sham (P < 0.01). MDL-800 reduced NLRP3 (P < 0.01 vs. CCI). Adding ML385 raised NLRP3 back to levels that did not differ from the CCI group (P > 0.05). ELISA results for IL-1β and IL-18 appear in **Figure 7B**. CCI elevated both cytokines versus sham (P < 0.01). MDL-800 reduced both (P < 0.01 vs. CCI). Co-treatment with ML385 abolished this reduction, with levels statistically indistinguishable from CCI (P > 0.05 for each). Mechanical thresholds (PWT) at day 10 are depicted in **Figure 7C**. CCI reduced PWT versus sham (P < 0.01). MDL-800 elevated PWT (P < 0.01 vs. CCI). ML385 co-treatment reversed this effect, with thresholds not different from CCI (P > 0.05). Thermal latencies (PWL) appear in **Figure 7D**. CCI shortened PWL versus sham (P < 0.01). MDL-800 prolonged PWL (P < 0.01 vs. CCI). ML385 co-treatment returned PWL to CCI-like levels (P > 0.05). Across all parameters, ML385 treatment restored values to levels that did not differ from the CCI-only condition. Therefore, the analgesic, anti-inflammatory, and anti-inflammasome actions of SIRT6 activation require an intact Nrf2 signaling pathway.

## Discussion

The present investigation establishes that SIRT6 expression declines precipitously within spinal dorsal horns following peripheral nerve constriction, while pharmacological activation of this NAD+-dependent deacetylase utilizing either UBCS039 or MDL-800 significantly attenuates mechanical allodynia and thermal hyperalgesia in a rat neuropathic pain model (Jain et al., 2025). These behavioral improvements correlate tightly with restoration of antioxidant enzyme activities, suppression of lipid peroxidation, and diminished production of IL-1β and IL-18, implicating SIRT6 as a critical regulator of both oxidative homeostasis and neuroinflammatory responses within nociceptive circuits (Zhang et al., 2026). The observation that SIRT6 downregulation occurs specifically at day 10 post-injury suggests that this epigenetic modifier participates actively in chronic pain maintenance rather than merely representing a passive consequence of neuronal damage (Montazeri-Khosh et al., 2025). The mechanism by which CCI reduces SIRT6 expression warrants consideration. Peripheral nerve injury triggers neuroinflammation, and pro-inflammatory cytokines such as TNF-α and IL-1β can suppress sirtuin expression through NF-κB-mediated transcriptional repression. Oxidative stress and mitochondrial dysfunction may also accelerate SIRT6 protein degradation. While our study did not directly investigate these upstream mechanisms, future studies examining transcriptional and post-translational regulation of SIRT6 following nerve injury would be valuable (Montazeri-Khosh et al., 2025).

Prior investigations have documented robust activation of the NLRP3 inflammasome across diverse neuropathic pain models, including spinal nerve ligation and chemotherapy-induced peripheral neuropathy, establishing this multiprotein complex as a pivotal driver of central sensitization (Liu et al., 2020). Our findings demonstrating that SIRT6 activation reduces NLRP3, ASC, and cleaved caspase-1 protein abundance align mechanistically with recent reports showing that genetic or pharmacological inhibition of the inflammasome attenuates mechanical hypersensitivity in rodent models (Shao et al., 2021). However, whereas previous studies targeted NLRP3 directly utilizing MCC950 or genetic knockout approaches, our strategy of upstream modulation via SIRT6 represents a novel intervention point that potentially offers broader regulatory control over multiple inflammatory and oxidative pathways simultaneously (Montazeri-Khosh et al., 2025).

The Nrf2 antioxidant response pathway has emerged as a critical protective mechanism against chronic pain, with insufficient endogenous Nrf2 activation underlying the transition from acute nociceptive responses toward persistent neuropathic states (Wang et al., 2022). Our demonstration that SIRT6 activation restores Nrf2 and HO-1 expression following nerve injury extends previous observations from cerebral ischemia models, where SIRT6 overexpression enhanced Nrf2 nuclear accumulation and reduced oxidative stress in neuronal tissues (Hu et al., 2022). Notably, the complete reversal of SIRT6-mediated analgesic effects by ML385 co-administration confirms that Nrf2 activation constitutes an obligatory intermediate step in the antinociceptive mechanism, distinguishing this pathway from Nrf2-independent antioxidant effects observed with other sirtuin family members (Wang et al., 2023).

SIRT6 is expressed in both neurons and glial cells, and each population may contribute differentially to pain modulation (Chen et al., 2025). The relationship between SIRT6 and oxidative stress regulation has been characterized extensively in metabolic and cardiovascular disease contexts, where this deacetylase suppresses mitochondrial reactive oxygen production and attenuates cellular senescence through multiple mechanisms, including direct regulation of mitochondrial proteins and modulation of antioxidant transcriptional programs (Bernatoniene et al., 2026). Our observation that SIRT6 activation restores superoxide dismutase and glutathione peroxidase activities while reducing TBARS accumulation suggests that similar protective mechanisms operate within spinal nociceptive circuits, potentially explaining the broad-spectrum efficacy observed across both mechanical and thermal pain modalities (Shen et al., 2026). The convergence of SIRT6-mediated regulation on both antioxidant enzyme induction and inflammasome suppression provides a mechanistic basis for the superior efficacy observed compared to treatments targeting single downstream effectors (Akantibila and Carabetta, 2025).

Several limitations constrain the interpretation of these findings and warrant acknowledgment. First, the pharmacokinetic profiles of UBCS039 and MDL-800 within central nervous system tissues remain incompletely characterized, and the degree of spinal cord penetration achieved through systemic administration has not been directly quantified in this investigation. Although both compounds have demonstrated *in vivo* efficacy in peripheral tissues, direct measurements of drug concentrations in cerebrospinal fluid and spinal parenchyma—ideally using mass spectrometry-based pharmacokinetic analyses—are needed to establish dose-response relationships for central target engagement (Akantibila and Carabetta, 2025). Second, although our data establish Nrf2 dependence through pharmacological inhibition, the precise molecular mechanism linking SIRT6 activation to enhanced Nrf2 stability or transcriptional activity within spinal neurons and glia requires further elucidation through chromatin immunoprecipitation and protein interaction studies (Akantibila and Carabetta, 2025). Third, while our immunofluorescence data show SIRT6 expression in spinal dorsal horn neurons, we did not distinguish the specific contributions of different spinal cord cell types (neurons, microglia, astrocytes) to the observed effects. Recent evidence indicates SIRT6 is expressed in both neurons and glial cells, and each population may contribute differentially to pain modulation (Chen et al., 2025). Cell-specific conditional knockout models targeting SIRT6 deletion in spinal neurons, microglia, or astrocytes would clarify which cellular populations mediate the observed antinociceptive effects. Immunohistochemical co-localization studies with cell-type-specific markers (NeuN for neurons, Iba1 for microglia, GFAP for astrocytes) represent a more immediately feasible approach to determine SIRT6 expression patterns across cell types. Fourth, our experiments used only male rats, and whether the observed effects generalize to females remains unknown. Given recognized sex differences in neuropathic pain mechanisms, future investigations should examine SIRT6 activation in female subjects across diverse pain etiologies (Vanneste et al., 2026).

Future investigations should address these limitations through direct measurement of drug concentrations within cerebrospinal fluid and spinal parenchyma, ideally utilizing mass spectrometry-based pharmacokinetic analyses to establish dose-response relationships for central target engagement (Akantibila and Carabetta, 2025). Additionally, cell-specific conditional knockout models targeting SIRT6 deletion in spinal neurons, microglia, or astrocytes would clarify which cellular populations mediate the observed antinociceptive effects and whether non-neuronal cells contribute significantly to the inflammatory modulation observed (Bernatoniene et al., 2026). The development of more potent and brain-penetrant SIRT6 activators with optimized pharmacokinetic properties represents a critical translational priority, as current compounds may possess limited clinical utility due to suboptimal central nervous system bioavailability (Akantibila and Carabetta, 2025). Finally, examination of SIRT6-mediated effects in female subjects and across diverse pain etiologies, including diabetic neuropathy and chemotherapy-induced peripheral neuropathy, would establish the generalizability of these findings to the broader neuropathic pain population (Vanneste et al., 2026).

## Conclusion

SIRT6 activation ameliorates neuropathic pain behaviors through Nrf2-dependent suppression of spinal NLRP3 inflammasome activation and restoration of antioxidant defenses. This investigation identifies SIRT6 as a previously unrecognized therapeutic target for neuropathic pain and establishes a novel SIRT6-Nrf2-NLRP3 signaling axis within spinal nociceptive processing.

## Data Availability

The raw data supporting the conclusions of this article will be made available by the authors, without undue reservation.
