# Correction: SIRT6 activation relieves neuropathic pain by restoring Nrf2 signaling and inhibiting NLRP3 inflammasome

**DOI:** 10.3389/fphys.2026.1974504

**Published:** 2026-09-08

**Authors:** Xintong Hao, Junkang Leng, Jianjun Tan

**Affiliations:** 1 Wuhan No 1 Hospital, Wuhan, China; 2 General Hospital of The Yangtze River Shipping, Wuhan Brain Hospital, Wuhan, China

**Keywords:** neuropathic pain, NLRP3 inflammasome, Nrf2 signaling, oxidative stress, SIRT6

Author “Junkang Leng” was erroneously omitted as equal contributing author.

The original version of this article has been updated.

